# Limited Short-Term Evolution of SARS-CoV-2 RNA-Dependent RNA Polymerase under Remdesivir Exposure in Upper Respiratory Compartments

**DOI:** 10.3390/v16101511

**Published:** 2024-09-24

**Authors:** Vladimir Novitsky, Curt G. Beckwith, Kristin Carpenter-Azevedo, Jimin Shin, Joel Hague, Soya Sam, Jon Steingrimsson, Richard C. Huard, Kevin Lethbridge, Sujata Sahu, Kim Rapoza, Karen Chandran, Lauri Bazerman, Evelyn Hipolito, Isabella Diaz, Daniella Carnevale, August Guang, Fizza Gillani, Angela M. Caliendo, Rami Kantor

**Affiliations:** 1 Department of Medicine, Warren Alpert Medical School, Brown University, Providence, RI 02912, USA; vnovitsky@lifespan.org (V.N.); cbeckwith@lifespan.org (C.G.B.); jhague@lifespan.org (J.H.); ssam2@lifespan.org (S.S.); fgillani@lifespan.org (F.G.); angela.caliendo@brownphysicians.org (A.M.C.); 2 State Health Laboratories, Rhode Island Department of Health, Providence, RI 02912, USA; kristin.azevedo@health.ri.gov (K.C.-A.); richard.huard@health.ri.gov (R.C.H.); 3 School of Medicine, University of Connecticut, Farmington, CT 06030, USA; jimin_shin@alumni.brown.edu; 4 Department of Biostatistics, School of Public Health, Brown University, Providence, RI 02912, USA; jon_steingrimsson@brown.edu; 5 Rhode Island Hospital, Providence, RI 02912, USA; klethbridge@lifespan.org (K.L.); ehipolito@lifespan.org (E.H.); idiaz2@lifespan.org (I.D.); dcarnevale3@lifespan.org (D.C.); 6 The Miriam Hospital, Providence, RI 02912, USA; sujata.sahu@lifespan.org (S.S.); krapoza1@lifespan.org (K.R.); kchandran@lifespan.org (K.C.); lbazerman@lifespan.org (L.B.); 7 Computational Biology Core, Center for Computation and Visualization, Brown University, Providence, RI 02912, USA; august_guang@brown.edu

**Keywords:** SARS-CoV-2, RdRp, remdesivir, respiratory compartments, drug resistance

## Abstract

Background: The extent of the SARS-CoV-2 short-term evolution under Remdesivir (RDV) exposure and whether it varies across different upper respiratory compartments are not fully understood. Methods: Patients hospitalized for COVID-19, with or without RDV therapy, were enrolled and completed up to three visits, in which they provided specimens from four respiratory compartments. Near full-length genome SARS-CoV-2 sequences were obtained from viral RNA, standard lineage and variant assignments were performed, and viral mutations in the RNA-dependent RNA polymerase (RdRp) region—the RDV target gene—were detected and compared between participants with and without RDV, across the four compartments, within participants across visits, and versus a larger sequence dataset. The statistical analysis used a generalized linear mixed-effects model. Results: A total of 139 sequences were obtained from 37 out of the 44 (84%) enrolled participants. The genotyping success varied across respiratory compartments, which ranged from 42% with oropharyngeal specimens to 67% with nasopharyngeal specimens and showed improvement with higher viral loads. No RdRp mutations known to be associated with RDV resistance were identified, and for 34 detected mutations at 32 amino acid positions that are not known as RDV-associated, there was no evidence of any associations with the RDV exposure, respiratory compartment, or time. At least 1 of these 34 mutations were detected in all participants, and some differed from the larger sequence dataset. Conclusions: This study highlighted the SARS-CoV-2 short-term genomic stability within hosts and across upper respiratory compartments, which suggests a lack of evolution of RDV resistance over time. This contributes to our understanding of SARS-CoV-2 genomic dynamics.

## 1. Introduction

Globally, a total of 775,552,205 confirmed COVID-19 cases (103,436,829 in the USA) and 7,050,201 COVID-19-associated deaths (1,188,195 in the USA) were reported to the World Health Organization by 26 May 2024 [1,2]. The public health response to the COVID-19 pandemic continues to evolve, which is leading to a substantial reduction in the risk of significant disease, hospitalization, and death from COVID-19 for most people [3].

Coronaviruses utilize an RNA-dependent RNA polymerase (RdRp) complex for the replication of their genome and the transcription of other viral genes [4,5,6,7]. The RdRp complex is the target of nucleoside analog inhibitors, such as remdesivir (RDV) [8,9,10,11]. RDV is a prodrug that inhibits viral RNA polymerases when metabolized intracellularly to an ATP analog [12,13]. RDV is inserted into RNA chains by RdRp during replication, which results in the premature termination of RNA synthesis and the inhibition of virus replication.

RDV is one of the few antivirals approved by the Food and Drug Administration for the treatment of COVID-19 [14,15]. The prophylactic and therapeutic effects of RDV against SARS-CoV-2 were demonstrated in hospitalized COVID-19 patients [11,12,16,17,18,19,20,21,22,23,24]. However, the potential emergence of RDV-associated resistance remains a concern for clinical care. In vitro studies showed the development of specific nonsynonymous mutations in RdRp (such as V166A, V166L, N198S, S759A, V792I, C799F/R, and E802D) after passaging SARS-CoV-2 in the presence of increasing concentrations of RDV or its analogs [25,26,27,28]. However, in vivo data on the development of RDV resistance in treated patients are limited. GISAID (the Global Initiative on Sharing All Influenza Data; https://gisaid.org/, accessed on 23 May 2023) exploratory analysis revealed very low levels of RDV resistance-associated mutations worldwide, despite the massive usage of this drug [26,29]. Analysis of 56,000 viral genomes from 105 countries detected negative selective pressure that affected nsp12 polymerase (non-structural protein 12, which is a component of the RdRp complex responsible for the viral replication and RNA synthesis), and suggested that RDV does not exert high selective pressure in the population [30]. The development of RDV-resistant mutations in the RdRp complex nsp7 (non-structural protein 7, which is another component of the RdRp complex essential for viral replication) and/or nsp12, such as D484Y, E802D, and V792I, was suggested in immunocompromised patients and recipients of organ and hematologic transplants on RDV therapy after prolonged infection [31,32,33,34,35]. It was also shown that RDV treatment in immunocompromised patients with prolonged infection can result in the rapid fixation of acquired mutations [36].

Recent studies in non-immunocompromised patients treated with RDV, including a phase 3 clinical trial, reported minimal intra-host variability up to 30 days after the initiation of RDV treatment, a negligible change in RDV susceptibility, and stochastic low-frequency variants, which suggests little selective pressure and a high barrier to RDV resistance [37,38]. At the same time, the extent and pace of SARS-CoV-2 genomic evolution under RDV selection pressure across upper respiratory compartments are not fully understood. Addressing this knowledge gap will inform the continued fight against COVID-19 and was the rationale for this study.

To assess the short-term SARS-CoV-2 RdRp evolution and determine whether it is influenced by RDV exposure across different upper airway anatomic locations, we genotyped SARS-CoV-2 that originated from four respiratory compartments of hospitalized patients in Rhode Island (RI), some of whom were treated with RDV. We considered three research areas: (i) whether short-term RDV exposure induces SARS-CoV-2 RDV-associated mutations in the RdRp region; (ii) whether there are inter-compartmental differences in RdRp mutations; and (iii) whether intra-host RdRp mutations accumulate over time. We also compared RdRp mutations in study participants with their prevalence among a wider RI dataset retrieved from GISAID. Better understanding of the emergence of SARS-CoV-2 RdRp mutations by RDV exposure across respiratory compartments and over time will inform the implementation of effective strategies for disease control and prevention.

## 2. Materials and Methods

### 2.1. Study Participants and Samples

Persons hospitalized with COVID-19 at the Miriam Hospital and RI Hospital in Providence, RI, between December 2020 and May 2022 were recruited for study participation. A subset of enrolled participants received up to 5 days of intravenous RDV, as determined by the clinical care providers. Each participant completed up to three study visits for respiratory specimen collection completed on separate days: baseline samples were obtained as soon as possible after hospitalization (before the first dose of RDV for participants who received RDV), with up to two additional specimen collections. Visits 2 and/or 3 were within 5 days after visit 1. The participants were offered to be sampled at each study visit from four respiratory compartments, including nasal (with standard foam swabs), nasopharyngeal (with a sterile dacron or nylon/rayon flocked swabs), oropharyngeal (with a sterile dacron or nylon/rayon flocked swabs), and saliva (with a DNA/RNA Shield saliva/sputum collection kit; Zymo Research, Irvine, CA, USA). The samples were collected by research assistants trained in standardized collection procedures. All swabs with specimens were placed into universal transport medium tubes with a sterile viral transport medium, while stabilizing solution was added to the saliva specimens. Demographic data, prior vaccination, and length of symptoms were collected from the participants and medical records. The study protocol was approved by The Miriam Hospital Institutional Review Board and all participants provided informed consent for study participation.

### 2.2. Viral Load Testing

Each collected specimen from each available compartment was tested for SARS-CoV-2 viral load. Viral RNA was isolated by the Qiagen EZ1 Advanced XL system (Qiagen, Germantown, MD, USA) and the Qiagen EZ1 DSP Virus Kit. The viral load was quantified using the ChromaCode HDPCR^TM^ assay (ChromaCode Inc., Carlsbad, CA, USA), which is a reverse transcription real-time polymerase chain reaction test calibrated to the WHO international standard for SARS-CoV-2 detection and quantification from upper/lower respiratory specimens. The association of viral RNA levels and successful genotyping was examined as outlined in the Section 2.7 below.

### 2.3. SARS-CoV-2 Genotyping

Viral genotyping was conducted using the same viral RNA employed for viral load testing for specimens with cycle thresholds (Ct) < 30. The viral RNA was reverse transcribed and amplified using the COVID-19 Artic v3 protocol [39]. Subsequently, the entire SARS-CoV-2 genome was sequenced by next-generation sequencing (NGS) on the Illumina MiSeq platform. Genomic analyses and quality control of generated SARS-CoV-2 sequences were conducted using publicly available tools and custom Python scripts, which are available under an open-source license from https://github.com/kantorlab/covid-pipeline, accessed on 23 May 2023). Single sequences per participant per compartment per study visit, with a length ≥ 25,000 bp, were included in the analyses.

### 2.4. SARS-CoV-2 Lineages and Variants

Lineage and variant assignments were performed using the pangolin and nextclade tools [40,41,42].

### 2.5. Definitions of RdRp Mutations

We analyzed the RDV-associated mutations in the SARS-CoV-2 RdRp region using the Stanford University Coronavirus Antiviral & Resistance Database [43]. Two groups of RdRp amino acid mutations were analyzed: mutations associated and not associated with RDV resistance. RdRp mutations associated with RDV resistance included 12 known mutations at 10 amino acid positions: V166L, A376V, F480L, D484Y, V557L, S759A, V792I, V796G, C799FR, and E802AD [25,27,36,38,43,44]. RdRp mutations not associated with RDV resistance were defined as differences from the wild-type virus (reference sequence NC045512.2; GenBank accession number MN908947) [45]. For the intra-host evolution analysis, amino acid mutations were defined as differences between the baseline and follow-up sequences for each person who had sequences available from multiple visits. The data on the global prevalence of analyzed mutations were derived from the Stanford University Coronavirus Antiviral & Resistance Database [43]. The prevalence of RdRp mutations identified in study participants was compared with the prevalence of corresponding mutations among available RI sequences retrieved from GISAID [46] that passed the quality control filters (https://github.com/kantorlab/covid-pipeline, accessed on 23 May 2023)), which was referred to as ‘the reference GISAID dataset’.

### 2.6. Analyses of RdRp Mutations

For the first research area (RDV exposure), we examined whether short RDV exposure induces RDV-associated mutations in the RdRp region. We used sequences only from the follow-up visits, not the baseline; grouped sequences from all compartments; and compared RdRp mutations between participants with and without RDV exposure.

For the second research area (compartments), we examined whether there were inter-compartmental differences in RdRp mutations. We grouped all sequences into four categories based on the compartment origin (nasal, nasopharyngeal, oropharyngeal, and saliva), regardless of the RDV exposure; assessed the presence of RdRp mutations; and compared mutations between different compartments while adjusting for the study visit and repeated measurement per participant.

For the third research area (intra-host evolution), we examined whether intra-host RdRp mutations accumulate over time. This analysis was performed only among participants who had sequences from multiple visits. We grouped all sequences regardless of the compartment or RDV exposure and assessed the evolution of RdRp mutations at the follow-up visits compared with the baseline sequences.

### 2.7. Statistical Analysis

To analyze the association between the viral load and the success of genotyping, we estimated the correlation coefficient and 95% confidence intervals. To compare the RdRp mutations between those who received RDV and those who did not (first research area), we employed a logistic mixed-effects model (LMM) with any mutation as the outcome, RDV as a covariate, and patient ID as a random intercept to account for the same participant having multiple study visits. Similarly, to compare RdRp mutations across compartments after adjusting for the study visit (second research area), we fitted an LMM with any mutation as the outcome, compartment and visits as covariates, and patient ID as a random intercept. Lastly, to compare RdRp mutations across study visits (third research area), we fitted an LMM with any mutation as the outcome, study visit as a covariate, and patient ID as a random intercept. A comparison of the prevalence of RdRp mutations identified in this study with the prevalence of corresponding mutations in the RI reference GISAID dataset was performed by using a test of proportions stratified by compartment and study visit [47]. Mutations with 0 or 100% prevalence in either study or reference GISAID dataset were excluded from the statistical tests due to a lack of variability, described without statistical comparison, and followed by a non-statistical statement regarding the observed differences. In all comparative analyses, the *p*-values were grouped by the type of analysis (either by compartment or by visit), and the Benjamini–Hochberg adjustment was applied to control for the within-group false discovery rate. All analyses were performed in R version 4.2.0 [48].

## 3. Results

### 3.1. Participants and Sampling

A total of 47 participants hospitalized with COVID-19 from two hospitals in RI were enrolled in the study. Three participants had undetectable viral loads in all collected specimens and were excluded (none received RDV). Among the remaining 44 participants (Table 1), 59% were males, with a median age of 59 years (range: 22 to 89 years old). A total of 75% were White or Caucasian and 14% were Hispanic or Latino, with a median of 6 (IQR 4–9) days since reported symptom onset, and 45% received at least one dose of the SARS-CoV-2 vaccine before hospitalization. The median SARS-CoV-2 viral load at the baseline visit was 6.14 log_10_ copies/mL (IQR 5.12–6.76). Following the baseline specimen collection, 18/44 participants received RDV intravenously, with a mean treatment length of 4 days (range: 1 to 5 days).

### 3.2. SARS-CoV-2 Genotyping

A total of 139 near-full-length genome SARS-CoV-2 sequences were obtained for 37 out of 44 (84%) study participants. The median sequence length was 29,751 bp (IQR 29,382–29,793 bp; range 25,289–29,829 bp). The median number of sequences per study participant was 4 (IQR 2–5; range 1–7), from a median of 4 (IQR 3–4; range 2–4) compartments per person. The successfully genotyped sequences were obtained from participants who completed from 1 to 3 study visits (median 2; IQR 1–2; range 1–3), including 38% (14/37) who completed one visit, 49% (18/37) who completed two visits, and 14% (5/37) who completed three visits. The median time between the first and last visits for participants with sequences was 2 days (IQR 1–2 days; range 1–4 days).

Successfully amplified and sequenced specimens are summarized by compartment and study visit in Table 2 (with further details in Supplementary Material Table S1). The genotyping success varied by respiratory compartment, which ranged from 42% (21/50) from collected oropharyngeal swabs in 31 participants to 67% (28/42) from collected nasopharyngeal swabs in 26 participants (oropharyngeal vs. nasopharyngeal: *p* < 0.05). As anticipated, the genotyping success was directly associated with higher levels of viral load (r = 0.66, 95% CI 0.58 to 0.73, *p* < 0.001).

### 3.3. SARS-CoV-2 Lineages and Variants

We identified 18 PANGO lineages assigned to six Spike variants, including a wild-type virus (4 participants, 3 lineages), Alpha (4 participants, 1 lineage), Delta (12 participants, 7 lineages), Epsilon (3 participants, 2 lineages), Iota (4 participants, 1 lineage), and Omicron (10 participants, 4 lineages; Table 3). The assigned lineages and variants were consistent across compartments and timepoints for each participant.

### 3.4. RdRp Mutations

**Figure 1:**
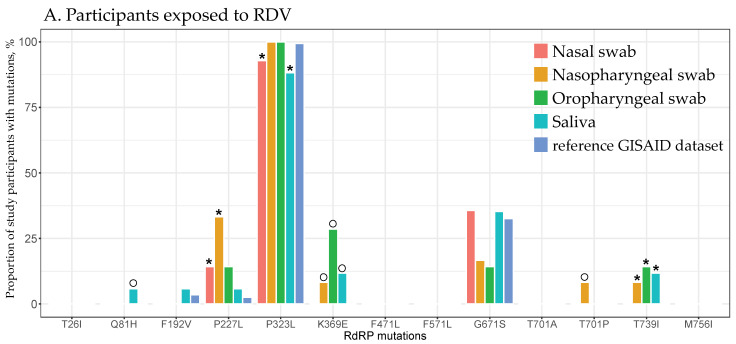

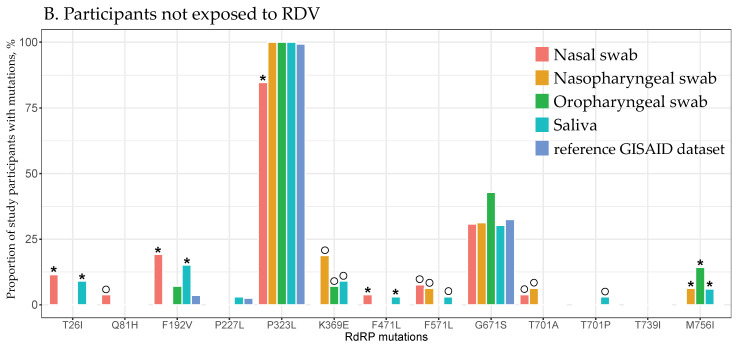
The distribution of RdRp mutations across the four upper respiratory compartments. Each respiratory compartment and the RI data from GISAID is represented by a different color, as indicated in the figure legend in the upper-right corner. The *x*-axis displays the RdRp mutations identified in multiple study participants, while the *y*-axis shows the proportion of study participants with identified RdRp mutations. The RdRp mutations are presented cumulatively across all study visits. Asterisks indicate statistically significant differences in the prevalence of mutations in specified compartments from the reference GISAID dataset. Open circles highlight the identified RdRp mutations in the specified compartments that were not present in the reference GISAID dataset. (**A**) Participants exposed to RDV. (**B**) Participants not exposed to RDV.

**Figure 2:**
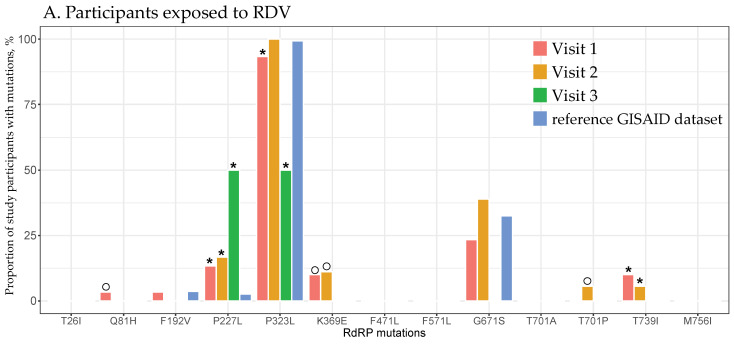

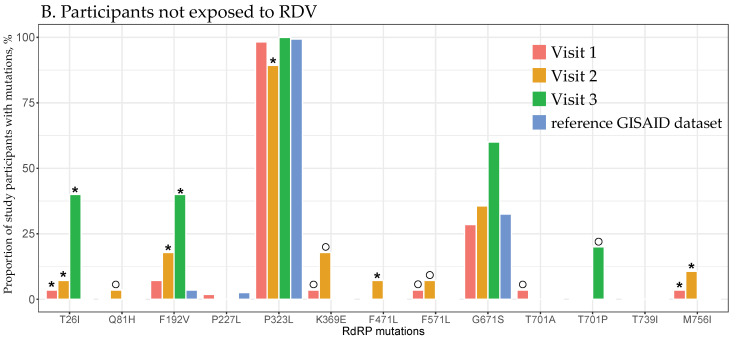
The distribution of RdRp mutations across study visits. Each visit and the RI data from GISAID is represented by a different color, as indicated in the figure legend at the upper-right corner. The *x*-axis indicates the RdRp mutations identified in multiple study participants, while the *y*-axis shows the proportion of study participants with identified RdRp mutations. The RdRp mutations are presented cumulatively across all four respiratory compartments. Asterisks indicate statistically significant differences in the prevalence of mutations at specified study visits from the reference GISAID dataset. Open circles highlight the identified RdRp mutations by study visit that were not present in the reference GISAID dataset. (**A**) Participants exposed to RDV. (**B**) Participants not exposed to RDV.

None of the 10 SARS-CoV-2 RdRp mutations previously associated with resistance to RDV [25,27,36,38,43,44] were identified among the study participants, regardless of the RDV exposure, compartment, or study visit. However, 34 mutations were identified at 32 amino acid RdRp positions, none of which are known to be associated with RDV resistance. At least one of these mutations was detected in all 44 participants, and 13 out of the 34 mutations, which spanned 12 amino acid positions, were found in more than one participant. The distributions of these mutations by RDV exposure across four respiratory compartments (cumulatively from all visits) and by study visit (cumulatively across all compartments) are shown in Figure 1 and Figure 2, respectively.

The testing of the first research area (RDV association) was limited to 23 participants with available sequences at follow-up visits, which involved 9 with and 14 without RDV exposure. None had RDV-associated mutations, as noted above, and all 23 had at least one of the 34 other RdRp mutations in the follow-up sequences (grouping all available sequences from all compartments). We found no statistically significant difference in the presence of RdRp mutations between the participants that received and did not receive RDV.

The testing of the second research area (compartment associations) included all sequences from all participants (Table 2). Non-RDV-associated mutations in RdRp were found in 24/25, 13/13, and 2/2 nasal sequences from the first, second, and third visits; 18/18, 9/9, and 1/1 nasopharyngeal sequences; 13/13, 8/8, and 0/0 oropharyngeal sequences; and 30/30, 16/16, and 4/4 saliva sequences. We found no statistically significant difference in the presence of RdRp mutations between the four respiratory compartments.

The testing of the third research area (intra-host evolution) was limited to 21 of 37 study participants with available sequences from at least two study visits, with 8 nasopharyngeal, 4 oropharyngeal, 13 nasal, and 15 saliva specimens. Grouping all compartments together, new RdRp mutations were observed only in the follow up compared with baseline sequences within each participant in 6/21 (29%) in the second visit and 1/21 (5%) in the third visit. We found no statistically significant difference in the presence of RdRp mutations across the study visits. We observed multiple acquired and lost mutations in the RdRp region that were specific to each participant, without any discernible pattern.

The prevalence of identified RdRp mutations among the participants in this study differed from the prevalence of some corresponding mutations among the 25,608 sequences from RI in the reference GISAID dataset (Figure 1). Among the participants exposed to RDV, the probability of the P227L mutation in the nasal and nasopharyngeal specimens and T739I in the nasopharyngeal, oropharyngeal, and saliva specimens was significantly higher, while the probability of P323L in the nasal and saliva specimens was significantly lower compared with the reference GISAID dataset (all adjusted *p*-values < 0.05; Figure 1A, Supplementary Material Table S2). Among the participants not exposed to RDV, the probabilities of T26I, F192V, and F471L in the nasal and saliva specimens, as well as M756I in nasopharyngeal, oropharyngeal, and saliva specimens, were significantly higher, while the probability of P323L in the nasal specimens was significantly lower compared with the reference GISAID dataset (all adjusted *p*-values < 0.05; Figure 1B, Supplementary Material Table S2).

Among the participants exposed to RDV, the probability of P227L at all three visits and T739I at the first and second visits was higher, whereas the probability of P323L at the first and third visits was lower than in the reference GISAID dataset (all adjusted *p*-values < 0.05; Figure 2A, Supplementary Material Table S3). Among those who did not receive RDV, the probabilities of T26I at all three visits, F192V at the second and third visits, F471L at the second visit, and M756I at the first and second visits were higher, while the probability of P323L at the second visit was lower compared with the reference GISAID dataset (all adjusted *p*-values < 0.01; Figure 2B, Supplementary Material Table S3).

Among the participants with sequences available from multiple specimen collections, we identified eight mutations (K369E in three participants; F471L and F571L in two participants each; and Q81H, D684V, A685P, T686Q, and T701P in one participant each) that were not present at the baseline visit but appeared at follow-up visits.

Lastly, some RdRp mutations were seen at extremely low prevalences in the reference GISAID RI dataset as compared with some compartments and study visits of participants (<0.01%; Figure 1 and Figure 2), e.g., Q81H, K369E, and T701P among participants exposed to RDV and Q81H, K369E, F571L, and T701A/P among those who did not receive RDV.

## 4. Discussion

The COVID-19 pandemic has had a profound impact on global health. Despite the use of several SARS-CoV-2-specific medications, such as RDV, our therapeutic arsenal remains limited. The emergence of RDV resistance could compromise its efficacy and future treatment options; however, the likelihood of RDV resistance remains uncertain and its study has been limited. Furthermore, given that samples can be obtained from various upper respiratory anatomical locations, it is unclear whether there are compartment-specific differences in viral evolution or whether short-term evolution occurs independently of RDV treatment. In this study, we did not observe the short-term evolution of SARS-CoV-2 RdRp RDV-associated mutations, or inter-respiratory-compartmental genomic differences, and found only stochastic intra-host viral evolution. These findings are encouraging and support continued RDV short-term use and the ability to monitor changes in viral evolution from multiple upper respiratory compartments.

Our finding that no RDV-associated resistance mutations developed in four upper respiratory compartments over short-term (up to 5 days) RDV use verified the high barrier to RDV resistance, as suggested by previous studies [37,38]. This outcome is not surprising given the short treatment course and the fact that participants were not immunocompromised (assumed and not directly assessed here), a population in which resistance development may be more common [32,49]. This finding remains reassuring and supports the continued use of RDV by suggesting that its short-term treatment is unlikely to be compromised by the development of drug resistance.

Investigating RdRp diversity remains essential to understanding viral evolution and adaptation. While previous studies found that the number of mutations, including in the RdRp region, positively correlated with the disease severity, and that specific mutations were associated with clinical outcomes and elevated mortality [50,51,52], comparative analyses of RdRp diversity across different respiratory compartments are limited. In this study we characterized viral RdRp diversity across four upper respiratory compartments and demonstrated overall genomic similarity. If RdRp genotyping success and outcomes are not affected by the origin of the sample, this suggests flexibility in the sampling strategy for SARS-CoV-2 genotyping, at least for RdRp. This methodological flexibility implies that samples from the different upper respiratory compartments can be used interchangeably for RdRp genotyping without compromising the accuracy or consistency of the results. Such interchangeability of respiratory compartments can reduce the logistical constraints and potentially increase the accessibility and efficiency of large-scale SARS-CoV-2 molecular studies. Moreover, our results confirm the robustness of RdRp genotyping regardless of variations in sample collection methods, which can simplify sample collection according to individual patient preferences and facility capacity and eliminate the need for sampling from multiple respiratory compartments.

While there was no evidence that the identified RdRp mutations in this study were clinically relevant, at least 1 of the 34 mutations at 32 RdRp positions were detected in all participants across RDV exposure, compartments, and study visits, and some differed from a larger comparator RI dataset. Out of these 34 mutations, only 2 (P227L and P323L) were also among 38 RdRp mutations identified by Hedskog et al. [38], and none matched the 31 RdRp mutations reported by Nirmalarajah et al. [37], thus further questioning their significance and suggesting the need for further research. Notably, few mutations were rare or did not occur and significantly differed from the reference RI GISAID dataset. Though we had small numbers of available genotypes, particularly when broken down by RDV, compartment, and study visit, this was an unexpected observation in such a small dataset when compared with a much larger one. It is worth mentioning that while sequences with these rare mutations passed quality control, some coverage over the RdRp regions containing these mutations was on the lower end. Taken together, these findings require further study to assess their potential relevance. They highlight the importance of genotyping surveillance to monitor the prevalence of RdRp and other gene mutations and their potential association with clinical outcomes, which ensures more effective management and clinical care of SARS-CoV-2 infections.

SARS-CoV-2 genotyping in this study correlated with higher levels of the SARS-CoV-2 viral load. Successful amplification requires a critical mass of intact viral RNA, and amplicons cannot be obtained if the viral load is too low. SARS-CoV-2 viral load quantification is a more involved and less typically performed measurement assay as compared with the Ct value, which is more accessible and commonly used to assess the viral burden in clinical care and research, and was correlated with epidemiological trends and mortality [53,54]. While SARS-CoV-2 viral load measurements are likely more standardized than Ct value quantification, a strong inverse correlation between the Ct and viral load values in the quantification of SARS-CoV-2 is well established [55]. Whether genotyping success is also correlated with Ct values was not directly examined here and should be further explored.

Similar to the lack of development of RDV-associated mutations, the analysis of intra-host SARS-CoV-2 evolution over time revealed no significant viral evolution in the RdRp region. This finding can be explained by the reasonable assumption that none of the study participants were immunocompromised, as well as the short follow-up time [31,32,33,34,35,36]. The limited evolution in the RdRp region provided important insight into understanding the stability of the SARS-CoV-2 RdRp region during a short-term course of RDV treatment, which contributed to its high resistance barrier.

Limitations of this study included a relatively small sample size; limited availability of samples from respiratory compartments; even fewer longitudinal samples from each respiratory compartment; varied sequence coverage; a short follow-up period due to the 5-day RDV course; a focus exclusively on the nsp12 RdRp region, which is the primary RDV target; and the lack of standardization for specimen collection and testing. Therefore, the results should be interpreted with caution and larger studies are warranted. Despite these limitations, this study reinforced some concepts with the limited available data and provided valuable information on RDV resistance, compartment diversity, and viral evolution.

## 5. Conclusions

Our investigation of short-term SARS-CoV-2 RdRp evolution in hospitalized COVID-19 patients suggested no emergence of RDV-associated resistant mutations, overall genomic similarity across respiratory compartments, and limited intra-host evolution. These findings imply a high resistance barrier to short-term RDV therapy, insinuate the absence of compartment-specific mutation evolution with or without RDV exposure, and highlight the SARS-CoV-2 short-term genomic stability in the RdRp region.

## Figures and Tables

**Table 1:** Cohort characterization.

Variable		All Study Participants (*n* = 44)			Participants Exposed to RDV (*n* = 18)		Participants Not Exposed to RDV (*n* = 26)	
		Total, with and without Sequences	Genotyped	Not Genotyped	Genotyped	Not Genotyped	Genotyped	Not Genotyped
**Gender**	Males, n (%)	26 (59%)	23 (62%)	3 (43%)	13 (87%)	2 (67%)	10 (45%)	1 (25%)
	Females, n (%)	18 (41%)	14 (38%)	4 (57%)	2 (13%)	1 (33%)	12 (55%)	3 (75%)
**Median (IQR) age, years**		59 (52–73)	67 (53–73)	54 (45–57)	67 (59–77)	54 (50–54)	57 (49–73)	52 (39–63)
**Race**	White or Caucasian, n (%)	33 (75%)	29 (78%)	4 (57%)	15 (100%)	2 (67%)	14 (64%)	2 (50%)
	Black or African Americans, n (%)	6 (14%)	5 (14%)	1 (14%)	0 (0%)	0 (0%)	5 (23%)	1 (25%)
	Other, n (%)	5 (11%)	3 (8%)	2 (29%)	0 (0%)	1 (33%)	3 (14%)	1 (25%)
**Ethnicity**	Not Hispanic or Latino, n (%)	38 (86%)	33 (89%)	5 (71%)	15 (100%)	2 (67%)	18 (82%)	3 (75%)
	Hispanic or Latino, n (%)	6 (14%)	4 (11%)	2 (29%)	0 (0%)	1 (33%)	4 (18%)	1 (25%)
**Prior vaccine**		20 (45%)	19 (51%)	1 (14%)	6 (40%)	0 (0%)	13 (59%)	1 (25%)
**Median (IQR) days since disease onset**		6 (4–9)	6 (4–9)	6 (6–15)	9 (5–11)	15 (11–15)	6 (4–8)	6 (5–10)
**Median (IQR) number of compartments**		3 (2–4)	4 (3–4)	3 (2–3)	3 (2–4)	3 (3–3)	4 (3–4)	3 (2–3)
**Median (IQR) number of visits**		2 (1–2)	2 (1–2)	2 (1.5–3)	2 (2–3)	2 (2–2.5)	2 (1–2)	2 (1–3)
**Median (IQR) viral load at visit 1, log_10_ copies/mL**		6.14 (5.12–6.76)	6.26 (5.55–6.76)	4.46 (4.32–4.72)	5.94 (5.12–6.45)	4.32 (4.32–4.39)	6.55 (5.98–7.15)	4.72 (4.32–5.38)

Note: The percentages were calculated per variable per group (e.g., 59% for males was calculated as 26 males out of 26 males + 18 females = 44 participants in the total group with and without sequences).

**Table 2:** Collected specimens and NGS sequences per participant per compartment per study visit.

Variable	Compartment			
	Nasal Swab	Nasopharyngeal Swab	Oropharyngeal Swab	Saliva
Total number of collected samples per compartment	71	42	50	79
Genotyping sequences *, n (%)	40 (56%)	28 (67%)	21 (42%)	50 (63%)
Study visit 1 total sequences, n	25	18	13	30
Study visit 2 total sequences, n	13	9	8	16
Study visit 3 total sequences, n	2	1	0	4
Combined visits:				
Single visit only, n sequences	12	12	13	17
Study visit 1	12	11	9	15
Study visit 2	0	1	4	1
Study visit 3	0	0	0	1
Two-visit sequences (participants)	22 (11)	16 (8)	8 (4)	24 (12)
Study visits 1 + 2	22	14	8	24
Study visits 1 + 3	0	0	0	0
Study visits 2 + 3	0	2	0	0
Three-visit sequences (participants)	6 (2)	0	0	9 (3)

Note: * single sequence per participant per compartment per study visit.

**Table 3:** Identified PANGO lineages and variants.

Spike Variant	PANGO Lineage	Study Participants, n	Proportion, %
Alpha	B.1.1.7	4	10.8
Delta	AY.103	2	5.4
Delta	AY.119	1	2.7
Delta	AY.122	1	2.7
Delta	AY.25	1	2.7
Delta	AY.3	3	8.1
Delta	AY.44	3	8.1
Delta	AY.54	1	2.7
Epsilon	B.1.427	2	5.4
Epsilon	B.1.429	1	2.7
Iota	B.1.526	4	10.8
Omicron	BA.1.1	3	8.1
Omicron	BA.1.18	1	2.7
Omicron	BA.2	2	5.4
Omicron	BA.2.12.1	4	10.8
Wild type	B.1.2	1	2.7
Wild type	B.1.517	1	2.7
Wild type	B.1.637	2	5.4

## Data Availability

SARS-CoV-2 sequences used in this study, along with the accompanying metadata, were submitted to GISAID. The GISAID submissions are EPI_ISL_19420081 to EPI_ISL_19420218.

## Supplementary Materials

The following supporting information can be downloaded from https://www.mdpi.com/article/10.3390/v16101511/s1, Table S1: Breakdown of generated NGS sequences by participant, compartment, and number of study visits; Table S2: Comparison of RdRp mutations identified in the study by respiratory compartment and the reference GISAID dataset; Table S3: Comparison of RdRp mutations identified in the study by study visit and the reference GISAID dataset.
