# Supplementary material for: Limited Short-Term Evolution of SARS-CoV-2 RNA-Dependent RNA Polymerase under Remdesivir Exposure in Upper Respiratory Compartments
Source: Viruses. 2024 Sep 24;16(10):1511. doi: 10.3390/v16101511 (PMC11512361; doi:10.3390/v16101511)
Supplement: Supplementary file 1 [file viruses-16-01511-s001.zip › viruses-3205594-supplementary.pdf]

Supplementary Materials

Supplementary Table S1. Breakdown of generated NGS sequences by participant, compartment, and number of study visits.

Study ID	Total NGS sequences, n	Total completed visits, n	Max interval between visits, days	NGS sequences per participant per compartment				Days since onset to baseline	RDV exposure
				Nasal swab, n	Nasopharyngeal swab, n	Oropharyngeal swab, n	Saliva, n		
1001	7	2	3	1	2	2	2	9	Yes
2007	6	2	2	2	2	1	1	7	Yes
2002	5	2	3	2	2	1	0	2	Yes
2015	5	2	2	2	0	1	2	9	Yes
2022	5	2	2	2	2	0	1	3	Yes
1002	4	1	1	1	1	1	1	6	Yes
2001	4	2	3	0	1	1	2	6	Yes
2005	3	2	2	2	0	0	1	3	Yes
2020	3	2	2	1	0	0	2	15	Yes
2013	2	3	2	0	2	0	0	11	Yes
2023	2	1	1	1	0	0	1	9	Yes
2008	1	1	1	0	0	0	1	15	Yes
2021	1	1	1	0	0	0	1	10	Yes
2024	1	3	1	0	0	0	1	36	Yes
2027	1	1	1	0	0	0	1	0	Yes
1005	7	3	4	1	1	2	3	11	No
1008	6	3	4	3	0	0	3	2	No
1012	6	2	2	2	0	2	2	8	No
1013	6	3	3	3	0	0	3	4	No
2012	6	2	2	1	2	1	2	4	No
2030	6	2	2	2	2	0	2	6	No
2033	6	2	2	1	2	1	2	9	No
1006	5	2	2	2	1	1	1	3	No
1010	5	2	2	2	0	1	2	7	No
2019	5	2	2	0	1	2	2	0	No
1009	4	1	1	1	1	1	1	9	No
2006	4	1	1	1	1	1	1	5	No
2031	4	2	2	2	0	0	2	3	No
1011	3	2	3	1	0	0	2	6	No
2011	3	1	1	0	1	1	1	18	No
2026	3	1	1	1	1	0	1	6	No
2032	3	2	2	2	0	0	1	3	No
2009	2	1	1	1	1	0	0	4	No
2016	2	1	1	0	1	1	0	9	No
1003	1	1	1	0	0	0	1	6	No
2010	1	1	1	0	0	0	1	5	No
2018	1	1	1	0	1	0	0	4	No

Supplementary Table S2. Comparison of RdRp mutations identified in the study by respiratory compartment and the reference GISAID dataset.

RDV exposure	Compartment	Mutation	Probability difference	Low 95% CI	High 95% CI	Adjusted p-value*
Participants exposed to RDV	Nasal	P227L	0.117	0.014	0.374	**<0.05**
		P323L	-0.065	-0.308	-0.006	**<0.05**
		G671S	0.032	-0.162	0.287	0.896
	Nasopharyngeal	P227L	0.307	0.112	0.583	**<0.001**
		G671S	-0.158	-0.278	0.123	0.405
		T739I	0.082	0.014	0.353	**<0.001**
	Oropharyngeal	P227L	0.117	0.000	0.487	0.149
		G671S	-0.182	-0.300	0.188	0.489
		T739I	0.142	0.025	0.512	**<0.001**
	Saliva	F192V	0.023	-0.025	0.234	0.768
		P227L	0.033	-0.015	0.244	0.563
		P323L	-0.111	-0.337	-0.026	**<0.001**
		G671S	0.028	-0.152	0.262	0.896
		T739I	0.117	0.032	0.343	**<0.001**
Participants not exposed to RDV	Nasal	T26I	0.114	0.039	0.289	**<0.001**
		F192V	0.156	0.049	0.343	**<0.001**
		P323L	-0.147	-0.329	-0.055	**<0.001**
		F471L	0.038	0.007	0.189	**<0.001**
		G671S	-0.017	-0.160	0.175	0.904
	Nasopharyngeal	G671S	-0.013	-0.184	0.231	0.914
		M756I	0.062	0.011	0.283	**<0.001**
	Oropharyngeal	F192V	0.036	-0.023	0.279	0.631
		G671S	0.103	-0.111	0.349	0.563
		M756I	0.143	0.040	0.399	**<0.001**
	Saliva	T26I	0.090	0.030	0.235	**<0.001**
		F192V	0.116	0.031	0.273	**<0.01**
		P227L	0.004	-0.021	0.127	0.904
		F471L	0.030	0.005	0.153	**<0.001**
		G671S	-0.022	-0.152	0.148	0.896
		M756I	0.061	0.017	0.196	**<0.001**

Note: * the Benjamini-Hochberg adjusted p-values to control the false discovery rate.

Supplementary Table S3. Comparison of RdRp mutations identified in the study by study visit and the reference GISAID dataset.

RDV exposure	Visit	Mutation	Probability difference	Low 95% CI	High 95% CI	Adjusted p-value*
Participants exposed to RDV	1	F192V	-0.003	-0.030	0.131	0.941
		P227L	0.107	0.027	0.271	**<0.01**
		P323L	-0.060	-0.207	-0.012	**<0.01**
		G671S	-0.092	-0.207	0.084	0.378
		T739I	0.099	0.034	0.255	**<0.001**
	2	P227L	0.141	0.032	0.366	**<0.01**
		G671S	0.064	-0.122	0.289	0.642
		T739I	0.055	0.009	0.257	**<0.001**
	3	P227L	0.474	0.069	0.880	**<0.05**
		P323L	-0.493	-0.899	-0.088	**<0.001**
Participants not exposed to RDV	1	T26I	0.035	0.009	0.120	**<0.001**
		F192V	0.036	-0.008	0.134	0.238
		P227L	-0.008	-0.023	0.069	0.748
		P323L	-0.011	-0.088	0.003	0.393
		G671S	-0.039	-0.141	0.090	0.631
		M756I	0.036	0.010	0.121	**<0.001**
	2	T26I	0.070	0.019	0.226	**<0.001**
		F192V	0.143	0.043	0.320	**<0.001**
		P323L	-0.101	-0.265	-0.031	**<0.001**
		F471L	0.071	0.020	0.226	**<0.001**
		G671S	0.032	-0.118	0.217	0.748
		M756I	0.107	0.037	0.272	**<0.001**
	3	T26I	0.399	0.117	0.768	**<0.001**
		F192V	0.364	0.082	0.733	**<0.01**
		G671S	0.275	-0.094	0.557	0.297

Note: * the Benjamini-Hochberg adjusted p-values to control the false discovery rate.
